# A Pectin Polysaccharide from *Arnebia szechenyi* Kanitz and Its Digestion Product: Physicochemical Properties and Immunostimulatory and Antioxidant Activities

**DOI:** 10.3390/molecules30193852

**Published:** 2025-09-23

**Authors:** Surina Bo, Peng Zhao, Sarangua Ochir, Huiwen Pang, Mu Dan, Wenming Bai, Man Zhang, Jingkun Lu

**Affiliations:** 1 College of Pharmacy, Inner Mongolia Medical University, Jinshan Development Zone, Hohhot 010110, China; surinabo@immu.edu.cn (S.B.);; 2 Academy of Mongolian Medicine, Inner Mongolia Medical University, Jinshan Development Zone, Hohhot 010110, China; 3 Australian Institute for Bioengineering and Nanotechnology, The University of Queensland, Brisbane, QLD 4072, Australia; 4 Department of Chemistry, Science School, National University of Mongolia, Ulaanbaatar 976-13343, Mongolia; 5 College of Basic Medicine, Inner Mongolia Medical University, Jinshan Development Zone, Hohhot 010110, China

**Keywords:** *Arnebia szechenyi* Kanitz, pectin-like polysaccharide, structure elucidation, antioxidant activity, immunostimulatory activity

## Abstract

The root of *Arnebia szechenyi* Kanitz, known as “Mongolia Zicao,” has been widely used in traditional Chinese and Mongolia medicine. Herein, we aimed to characterize a pectin polysaccharide extracted from *A. szechenyi Kanitz* root (ASP), elucidate its structure, and evaluate potential immunomodulatory activities through in vitro assays. Sugar composition analysis and high-performance gel permeation chromatography (HPGPC) revealed that ASP is predominantly composed of GalA (45.44%), Gal (22.13%), and Ara (19.86%) with a homogenous molecular weight of 18.4 kD. ASP was identified as a typical pectin-like polysaccharide containing linear HG domains and potentially linked to complex branches with Ara and Glu residues. The monosaccharide analysis of the digestion product, D-ASP, supported this hypothesis. The Congo red test indicated the absence of a triple-helix structure in ASP and its digestion product D-ASP. ASP exhibited an irregular structure due to the branching fork, which disappeared after digestion, as observed by scanning electron microscopy. Additionally, ASP and D-ASP had certain antioxidant activities and significantly stimulated the release of cytokines (IL-1β, IL-6, TNF-a, NO), macrophage proliferation and phagocytic capability in RAW 264.7 cells in a dose-dependent manner. These findings outline the chemical and biological foundation for the development of novel drug candidates in the food and pharmaceutical industries.

## 1. Introduction

Zicao is a perennial herb in the family Boraginacese, first recorded in the ancient herbal classic *Shennong Ben Cao Jing*, and its roots have long been used as a medicinal agent in traditional Chinese medicine. The Zicao family comprises approximately 100 genera worldwide. In China alone, there are more than 200 families, 49 genera, and 208 species [1]. The Chinese Pharmacopoeia described medical Zicao as the dried root of *Arnebia euchroma* (Royle) Johnst (Xinjiang Zicao) and *Arnebia gutta* ta Bunge (Inner Mongolia Zicao) of the family Chinese Pharmacopoeia [2]. Xinjiang Zicao is the most common species currently found in herbal markets and pharmacies; however, the wild resources are severely damaged, and artificial cultivation has not yet succeeded. *Arnebia szechenyi* Kanitz, also known as “Mongolia Zicao,” belongs to the Boraginaceae family and is an herbaceous perennial plant that inhabits sunny rocky slopes and sand dune edges in western Inner Mongolia [3,4]. It is a unique and characteristic species of Zicao with a long history of medicinal use in traditional Mongolia medicine in the Inner Mongolia area, which is often used instead of Xinjiang Zicao in traditional Mongolian herbal formulations. Mongolia Zicao also contains active ingredients with antibacterial, heat-clearing, and blood-cooling effects [5]. *Arnebia szechenyi* Kanitz is frequently employed as a regional alternative. In addition to the known variations in active constituents such as shikonin derivatives, it is also worthwhile to investigate whether differences exist in polysaccharides, which represent another major class of active compounds.

Phytochemical research has revealed that the chemical compositions and pharmacological effects of shikonins found in Zicao species from Xinjiang and Mongolia are largely comparable [4]. While numerous plant-derived polysaccharides have been documented for their significant antitumor immune activities, the structure of polysaccharides from medicinal Zicao and their potential immunomodulatory and antitumor effects have received limited investigation. Previously, we investigated the structural properties and immunomodulatory effects of the ARP polysaccharide derived from the roots of *Arnebia euchroma* (Royle) Johnst (Xinjiang Zicao). It is suggested that ARP is likely an arabinogalactan, with a backbone consisting predominantly of Galp residues linked by 1,6-bonds, and Ara residues joined via 1,5 or 1,3 linkages. ARP significantly promoted the proliferation of B- and T-lymphocytes and increased the secretion of tumor necrosis factor-alpha (TNF-α), interleukin (IL)-6, and IL-1β, indicating that the polysaccharide induces the functional activation of macrophages. It is presumed that its outstanding vitro immunomodulatory activity was associated with its high content of arabinogalactan and unique structure [6].

Polysaccharides are natural macromolecular compounds found in plants, animals, and microorganisms. They display a range of pharmacological activities, including immunomodulation [7], antifibrotic [8], antiviral [9], antioxidant, antitumor [10], and hypoglycemic effects [11], as well as modulation of the gut microbiota [12,13]. Polysaccharides are abundant, safe, and offer great promise for development and application across various fields, including the food industry and biomedical sciences [14,15].

There is growing interest in investigating the potential applications of root polysaccharides from Mongolia Zicao. In this article, we have isolated and purified a novel pectin-like polysaccharide, named ASP, from the crude extract of *Arnebia szechenyi* Kanitz root. Furthermore, the fundamental structural features of ASP were characterized by monosaccharide composition, molecular weight distribution, methylation analysis, nuclear magnetic resonance (NMR) spectroscopy, Congo red test and scanning electron microscope (SEM), as well as its immunomodulatory and antioxidant properties in vitro. The target enzyme arabinosidase was selected to digest ASP and produced the digestion product D-ASP; then, the structure and activity was studied. The above results hopefully lay groundwork for future research into the relationship between the polysaccharide structure and biological activity.

## 2. Results

### 2.1. Isolation and Purification of Polysaccharides

**Figure 1:**
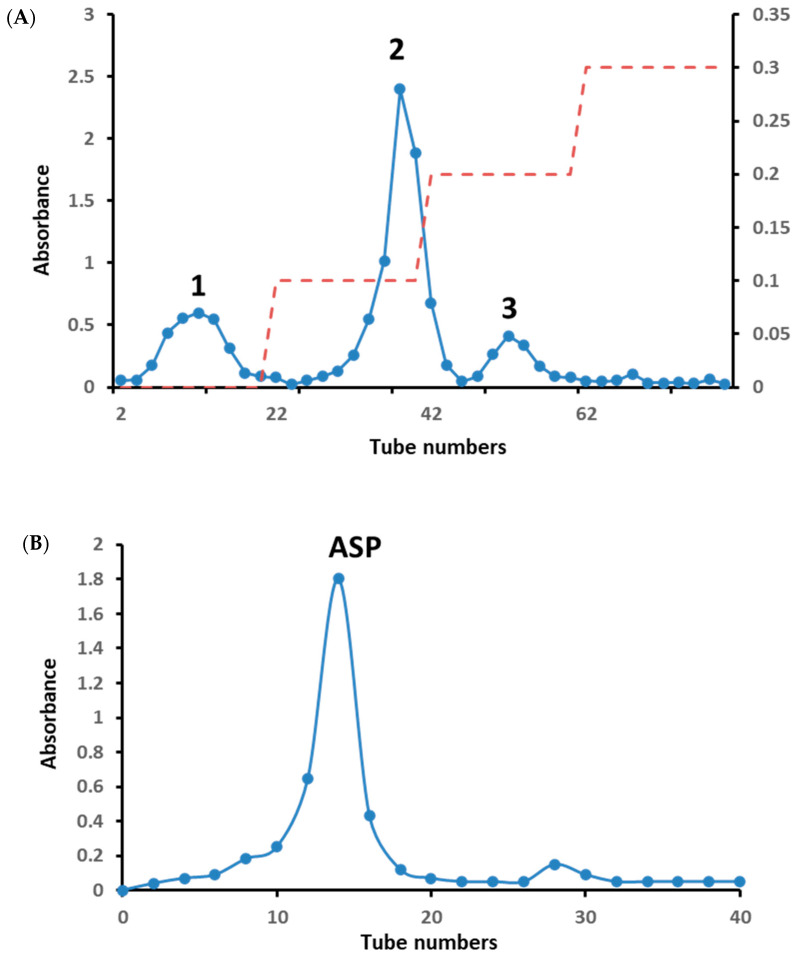

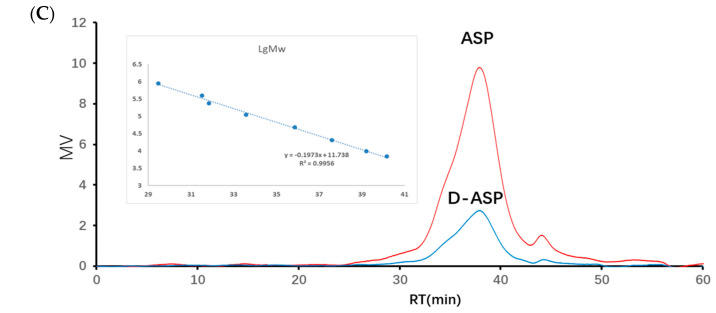
The elution curve of crude polysaccharide on DEAE-52 cellulose column with distilled water and 0.1–0.3 M NaCl (red dotted line) (**A**). The elution curve of peak-2 on Sephadex G-100 gel column (**B**). GPC chromatograph of ASP and D-ASP polysaccharides using GPC measured the molecular weights using HPGPC and the standard curve of dextran (**C**).

The crude polysaccharide extracted from the dry root yielded 6.04%. The three peaks 1, 2 and 3 were collected by a DEAE-52 cellulose column with distilled water and 0.1–0.3 M NaCl solution in Figure 1A, and the yields of the three peaks were calculated as 1.02 g (17.0%), 4.45 g (74.2%) and 0.86 g (14.3%), respectively. The amount of peaks 1 and 3 was low for no subsequent separation on a gel column. Peak 2 was further separated on a Sephadex G-100 gel column and the separation flow diagram, as shown in Figure 1B, yielded one homogeneous polysaccharide, named ASP. The total carbohydrate and protein content of ASP and digestion product D-ASP were determined to be 83.94 ± 1.03%, 91.24 ± 1.82% and 4.71 ± 0.43%, 3.71 ± 0.57%, respectively.

### 2.2. Molecular Weight Determination

The molecular weights (Mw) for ASP and D-ASP were found to be 1.84 × 10^4^ and 1.77 × 10^4^ Da, respectively, according to the standard dextran curve in Figure 1C. The digested polysaccharide exhibited a slightly lower molecular mass than ASP. This reduction can be attributed to the cleavage or fragmentation of the polysaccharide chain during the digesting process, leading to lower molecular weight fragments. Therefore, native polysaccharides have a larger molecular weight than digested derivatives, such as D-ASP.

### 2.3. Monosaccharide Composition Analysis

**Figure 2:**
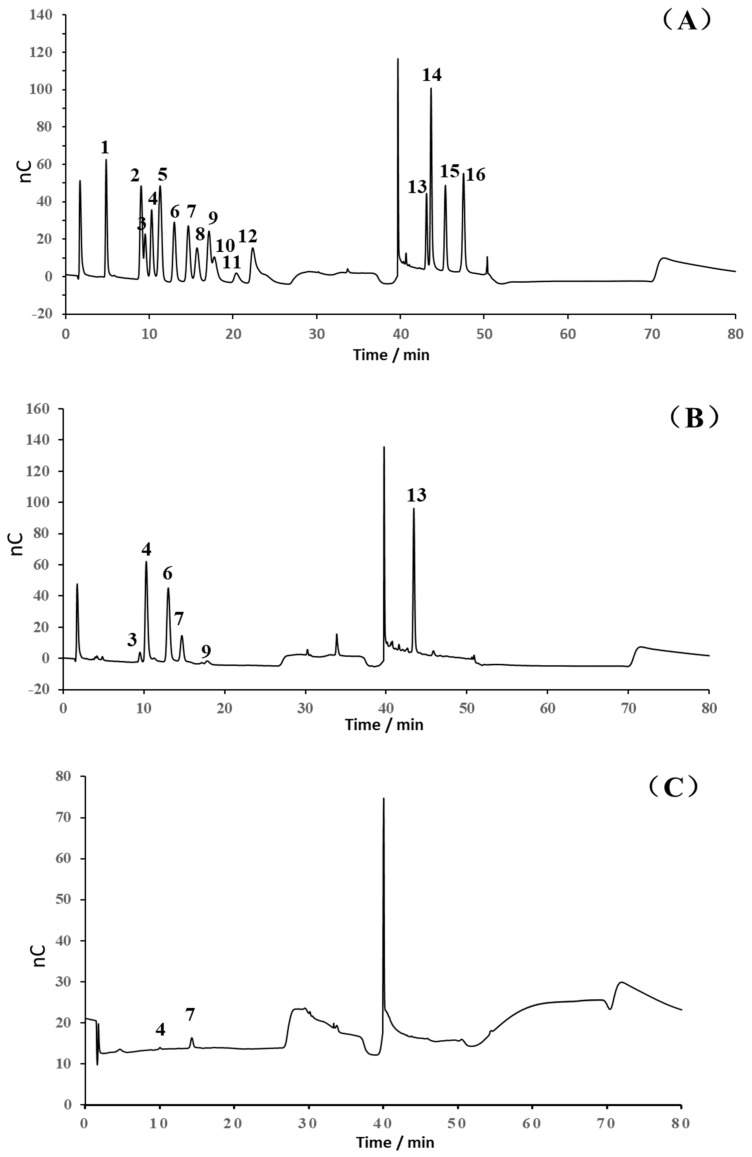
Monosaccharide composition analysis of ASP, (**A**) ion chromatography of standard monosaccharides, (**B**) ion chromatography of ASP, and (**C**) ion chromatography of digestion supernatant (1. Fuc, 2. GalN, 3. Rha, 4. Ara, 5. GlcN, 6. Gal, 7. Glu, 8. GlcNA, 9. Xyl, 10. Man, 11. Fru, 12. Rib, 13. GalA, 14. GulA, 15. GalA, and 16. ManA).

Based on the electrochemical activity of polysaccharides and their ionization characterization in strong alkaline solutions, high-performance anion-exchange chromatography (HPAEC) for the detection and quantitative analysis of 16 common monosaccharides was applied. This method utilized an anion-exchange column as the analytical column, an electrochemical detector, and a gradient elution manner. Compared with the traditional gas chromatography and high-performance liquid chromatography-1-phenyl-3-methyl-5-pyrazolone methods, the HPAEC with pulsed amperometric detection (PAD) offers the benefits of straightforward sample preparation and the capability to simultaneously analyze multiple monosaccharide components [16]. It is particularly effective for both the qualitative and quantitative analysis of glucuronic acid and amino sugars. The monosaccharide composition analysis of ASP revealed that it consisted of galacturonic acid, galactose, arabinose, glucose, rhamnose, and xylose in a molar ratio of 27.3:13.3:11.9:4.6:2.0:1 as shown in Figure 2B. ASP derived from Mongolia Zicao was identified as a heteropolysaccharide, primarily composed of galacturonic acid, galactose, and arabinose, with galacturonic acid (GalA) being the dominant component, accounting for 45.44%. This suggests that the linear chain of ASP is formed predominantly by GalA, corresponding to the homogalacturonan (HG) domain of pectin. Significant amounts of galactose (22.13%), arabinose (19.86%), and rhamnose (3.24%) indicated a major contribution from the rhamnogalacturonan-I (RG-I) domain. Additionally, the Rha/GalA ratio (2.0/27.3 = 0.07) confirms that the ASP backbone is predominantly composed of the HG domain rather than the RG-I type [17].

To further explore the relationship between structure and biological activity, an enzymatic hydrolysis experiment was performed. Only arabinose and glucose were detected in the digestion supernatant using a monosaccharide analysis in Figure 2C, which indicated that Glc and Ara were readily cleaved by enzymes in the peripheral branches. Enzyme digestion improved the solubility of ASP polysaccharides and produced specific structural fragments, which are referred to as D-ASP.

### 2.4. UV-Vis and FT-IR Spectrum

**Figure 3:**
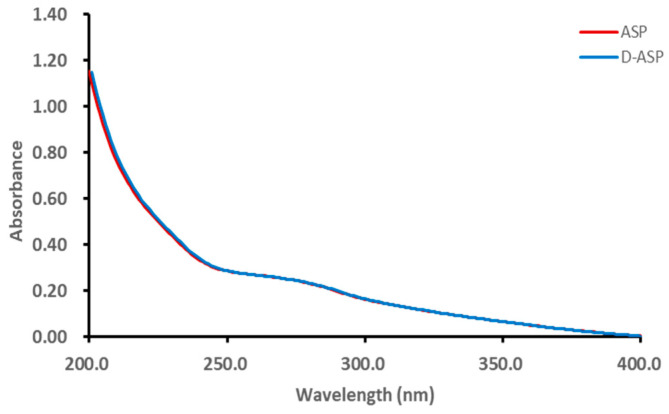
UV-Vis spectra of ASP and D-ASP polysaccharides.

As shown in Figure 3, it was suggested that the ASP and D-ASP fraction had low absorption at 260 or 280 nm in the UV spectra, indicating the low exitance of nucleic acid and protein.

**Figure 4:**
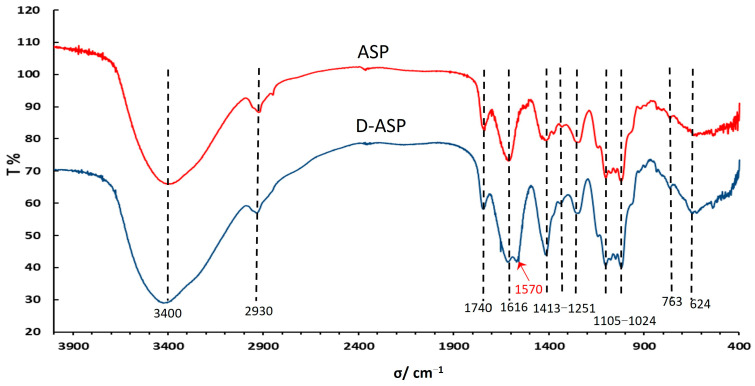
Fourier transform-infrared (FT-IR) spectra of ASP and D-ASP polysaccharides.

The FT-IR analysis results are depicted in Figure 4. The prominent and broad absorption peaks observed between approximately 3254 and 3406 cm^−1^ correspond to the stretching and deformation vibrations of the O–H group [18,19]. A peak around 2930 cm^−1^ is attributed to the stretching and bending vibrations of C–H bonds. Additionally, the peak near 1616 cm^−1^ represents the stretching vibration of the C=O group across all spectra [20]. The distinctive absorption peak at 1740 cm^−1^ is indicative of the COOH stretching vibration, confirming the presence of carboxyl groups in both ASP and D-ASP, implying that the enzymatic digestion process did not alter the uronic acids. Following enzyme digestion, new signals at 1570.06 and 1413.82 cm^−1^ were observed, corresponding to the asymmetric and symmetric vibrations of COO^−^, respectively, suggesting that glucuronic acid dissociated into carboxylate form during the enzymatic process [21]. Furthermore, the stretching vibrations of C–O–C and C–O–H contributed to absorption peaks within the range of 1105 to 1024 cm^−1^.

### 2.5. Methylation Analysis

As summarized in Table 1, ASP had 16 types of sugar residues, and the main sugar residue linkages were found to be Araf-(1→, →5)-Araf-(1→, →3,5)-Araf-(1→, →4)-Glcp-(1→, →4)-Galp-(1→, residues, which accounted for 70.50% among all sugar residues. This suggests that the backbone of ASP consists of four main sugar residues, while the branching points are mainly located at →3,5)-Araf-(1→ and →4)-GalAp-(1→. The combined molar percentages of terminal-linked sugar residues (0.157) were found to be comparable to those of branched sugar residues (0.102), which supported the validity of the methylation results. From the results of methylation and enzyme digestion experiments of ASP polysaccharide, branch sugar residues were speculated →3,5)-Araf-(1→, →3,4)-Glcp-(1→ and →4,6)-Glcp-(1→ and some terminal sugar residues Araf-(1→/Galp-(1→.

### 2.6. NMR Analysis of ASP

**Figure 5:**
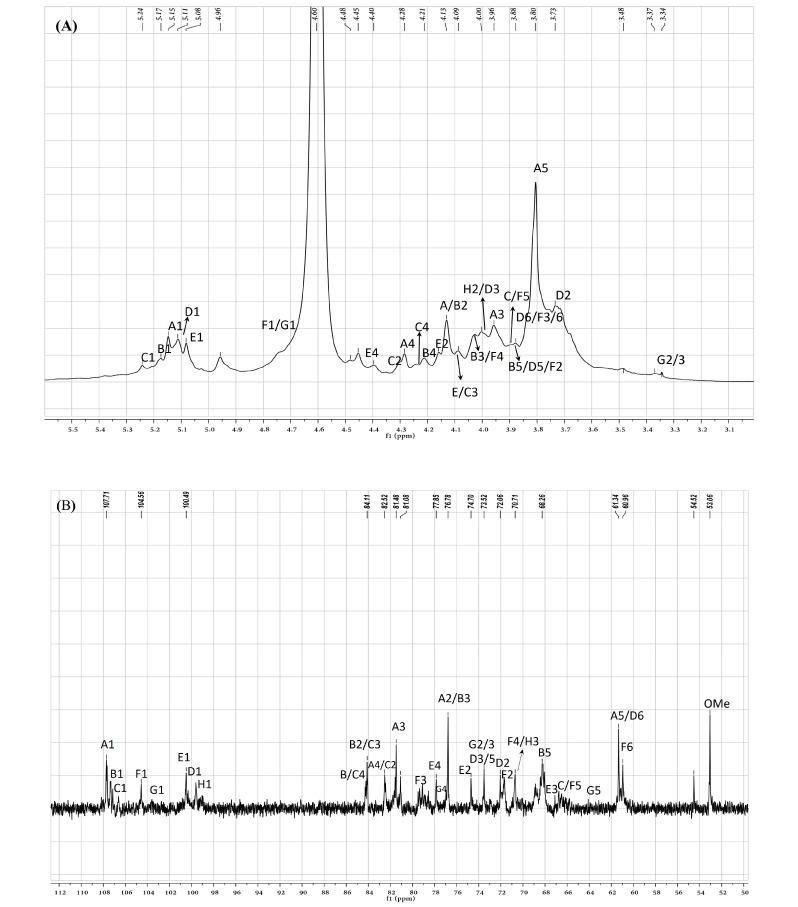

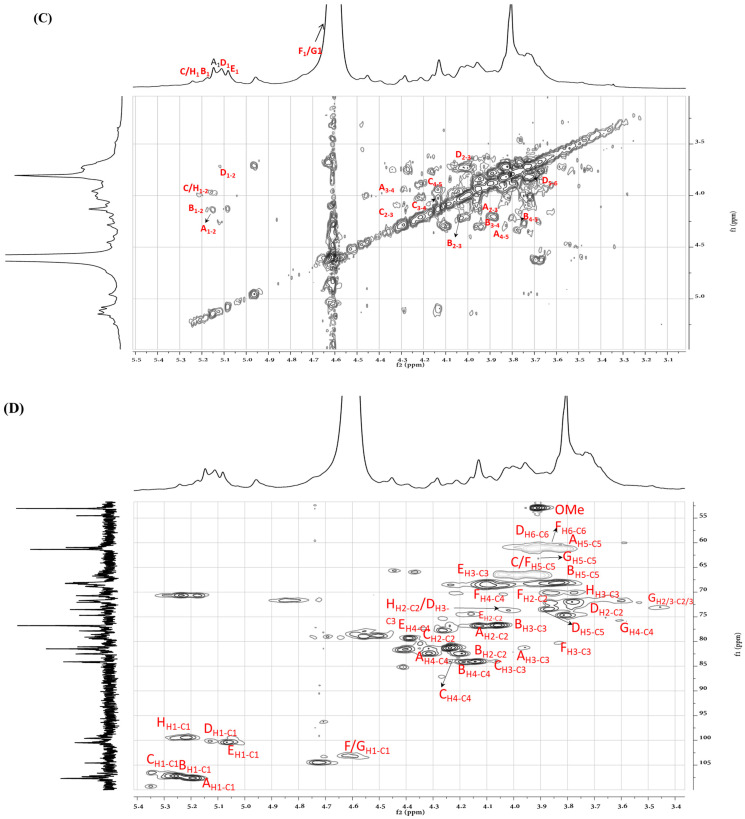

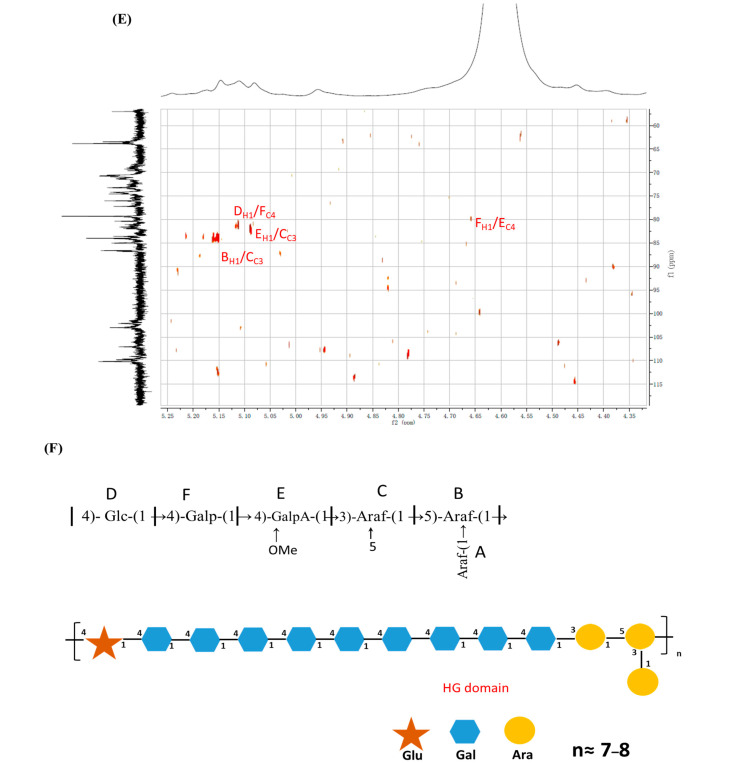
(**A**) ASP polysaccharide ^1^H NMR spectra, (**B**) ^13^C NMR spectra, (**C**) COSY spectra, (**D**) HSQC spectra, (**E**) HMBC and (**F**) the proposal structure fragment of ASP.

To further clarify the sequences of sugar residues in ASP polysaccharide, ^1^D NMR (^1^H NMR, ^13^C NMR) and ^2^D NMR (COSY, HSQC, and HMBC) were analyzed to attribute all C/H chemical shifts combining the literature and are summarized in Table 2. As shown in Figure 5A,B, eight anomeric proton and carbon signals were anatomized. In the ^13^C NMR spectrum of ASP, the strong signals at 107.7, 107.3, and 107.1 ppm were attributed to Araf-(1→ (A), →5)-Araf-(1→ (B), and →3,5)-Araf-(1→ (C) residues, respectively. The signals in the anomeric region of the ^1^H NMR spectrum of ASP (Figure 5A) were assigned based on the COSY (Figure 5C) and HSQC spectrum (Figure 5D). Signals at 5.14, 5.17, and 5.24 ppm were allocated to H1 of Araf-(1→ (A), →5)-Araf-(1→ (B), and →3,5)-Araf-(1→ (C) residues corresponding to the C-1 signals at 107.7, 107.3, and 107.1 ppm, respectively. In the range of δ 104–103 ppm, the signals at 103.1 and 103.2 ppm belonged to →4)-Galp-(1→ (F) and →4)-Xylp-(1→ (G) sugar residues. The signals at 4.78 and 4.58 ppm were set to H-1 of →3)-Galp-(1→ and →4)-Xylp-(1→ corresponding to the C-1 signal at 104.5 and 103.2 ppm. At the district of 100 ppm, two signals at 100.5 and 100.2 were assigned to 1→4)-GalpA-(6-OMe)-(E) and →4)-Glcp-(1→ (D, corresponding to the H-1 signals at 5.03 and 5.12 ppm, respectively. For the residue of Araf-(1→ (residue A), the starting resonance was H1 (5.14 ppm); then, the chemical shifts of H2 (4.13 ppm), H3 (3.96 ppm), H4 (4.23 ppm), and H5 (3.80 ppm) were all labeled in the homonuclear correlation spectroscopy spectrum (Figure 5C) corresponding to C2–5 signals that were appointed in the HSQC spectrum (Figure 5D). All the other signals are listed in Table 2. Featured C/H signals of the groups CH_3_COO–, MeO–, and MeC appeared at 20.3/2.18, 53.03/3.88, and 16.7/1.35 ppm [21]. The low-field signal at 171.0 ppm in the ^13^C NMR spectrum suggested the appearance of a carboxyl group in uronic acid.

The inter and intra-residues linkage information of ASP were further detected using the long-range heteronuclear multiple bond correlation (HMBC) spectrum. In the HMBC analysis of ASP (Figure 5E), cross-peaks were observed between H-1 of residue B and C-3 of residue C (B H-1/C C-3), indicating a connection between residues B and C. Similarly, cross-peaks were detected between H-1 of residue F and C-4 of residue E (E H-1/F C-4), suggesting a linkage between residues E and F. Additionally, a cross-peak between H-1 of residue E and C-3 of residue C (E H-1/C C-3) confirmed the connection between residues E and C. Moreover, a cross-peak was observed between H-1 of residue D and C-4 of residue F (D H-1/F C-4), which corroborated the linkage of residues D and F. The proposed primary sugar backbone of ASP is illustrated in Figure 5F. However, the linkage patterns for certain sugar residues (→4)-Xylp-(1→) remain ambiguous due to insufficient data from HMBC and NOESY spectroscopy.

### 2.7. Determination of a Triple-Helix Structure

**Figure 6:**
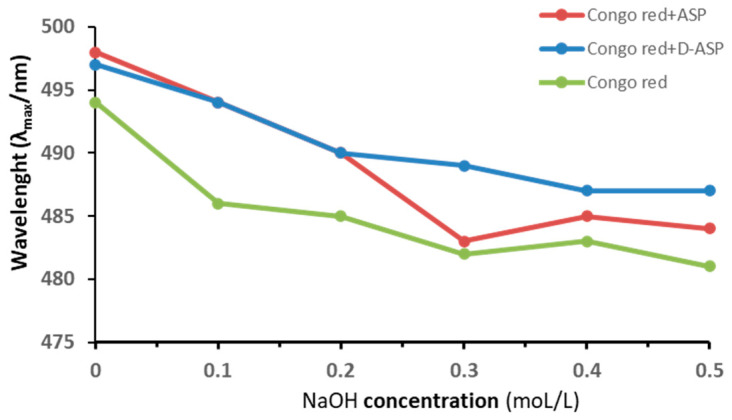
Maximum absorption wavelength of Congo red–polysaccharides (ASP and D-ASP) at various concentrations of sodium hydroxide for a triple-helical structure.

Congo red forms complexes with polysaccharides that possess a triple-helical structure, causing an increase in absorbance and a redshift in the Congo red at NaOH specific concentrations [19]. To investigate this, Congo red was reacted with APS polysaccharide and its digestion product D-ASP, and the shift in λ_max_ wavelength was measured at varying concentrations of NaOH in Figure 6. Neither the ASP nor the D-ASP polysaccharide-Congo red complex exhibited a significant red shift with increasing NaOH concentration, suggesting the absence of a triple-helix structure in both ASP and D-ASP.

### 2.8. Scanning Electron Microscope

**Figure 7:**
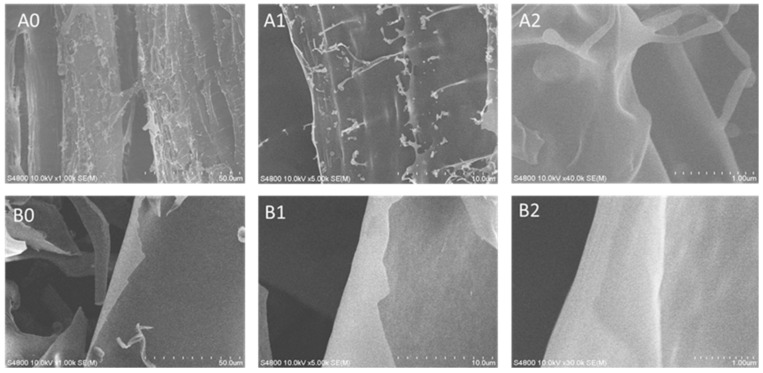
Scanning electron microscopy (SEM) images of ASP (**A0**–**A2**) and D-ASP (**B0**–**B2**). SEM images were captured at the magnifications of 100×, 500×, and 4000×, respectively. Left images: 100× magnification; middle images: 500× magnification; right images: 4000× magnification.

The surface morphologies of ASP and D-ASP were characterized using scanning electron microscopy. As shown in Figure 7, these polysaccharides exhibited distinct surface features. ASP possessed an irregular lamellar structure with many branches, superimposed parts in the structure, and rod-like particles attached to the surface of the lamellar structure. The solid structure reflects the interactions between polysaccharides in the liquid state, indicating that the ASP is an irregular structure formed through the interlocking entanglement of multiple structures. In contrast, D-ASP retained a lamellar structure, but showed a smoother and denser surface, with the most branching points disappeared. These observations suggest that the enzymatic digestion, primarily by removing arabinose residues, effectively cleaved nearly all the branches, including those formed by arabinose and glucose, which matches the result in monosaccharide composition of the digestion supernatant of ASP.

### 2.9. Antioxidant Activities of ASP and D-ASP

**Figure 8:**
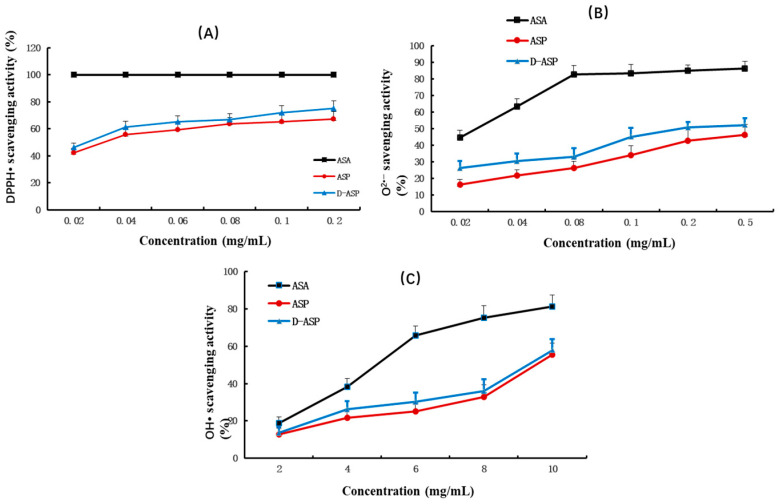
Antioxidant activity assay of ASP and D-ASP. (**A**) DPPH• radical scavenging assay; (**B**) superoxide anion scavenging activity assay; (**C**) hydroxyl radical scavenging ability assay; values are means x ± SD, *n* = 3.

The antioxidant effects of ASP and D-ASP were linked using an in vitro chemical model to scavenge chemically generated radicals, such as hydroxyl radical (OH•), superoxide (O^2•−^), and 2,2-diphenyl-1-picrylhydrazyl (DPPH•). The DPPH• assay is commonly employed as an initial screening technique for antioxidant activity owing to the nitrogen radical species’ remarkable stability, which is ascribed to the electron delocalization throughout the molecule. Figure 8A illustrates the DPPH• radical scavenging activity of ASP and its digested derivatives. D-ASP derivatives exhibited a much higher scavenging activity (46.10–75.12%) against the DPPH• radical than ASP (42.13–67.13%) with both polysaccharides displaying the IC_50_ values between 0.02 and 0.04 mg/mL. As shown in Figure 8B,C, both the superoxide anion and hydroxyl radical scavenging activities of ASP and D-ASP increased in a dose-dependent manner the tested concentration ranges (0.02–0.5 mg/mL for superoxide and 2.0–10.0 mg/mL for hydroxyl radicals). The D-ASP derivatives had slightly greater scavenging activity (ranging from 26.19% to 52.31%) against the superoxide anion radical compared with ASP, which displayed scavenging activity ranging from 16.19% to 46.31%. The IC50 for D-ASP polysaccharide was in the range of 0.2–0.5 mg/mL. ASP and D-ASP have similar hydroxyl radical scavenging ability with an IC_50_ of 8–10 mg/mL. These results indicate that D-ASP possesses marginally higher antioxidant activity compared to the native ASP, although the difference is not statistically significant. Zheng et al. revealed that the enzymatically hydrolyzed polysaccharide from Dandelion (*Taraxacum mongolicum*) demonstrated increased polysaccharide content and a lower molecular weight compared to the native polysaccharide. Furthermore, it exhibited enhanced antioxidant activity, including superior free radical scavenging capacity and greater reducing power compared to native polysaccharide [27].

### 2.10. Immunostimulatory Activities of ASP and D-ASP

**Figure 9:**
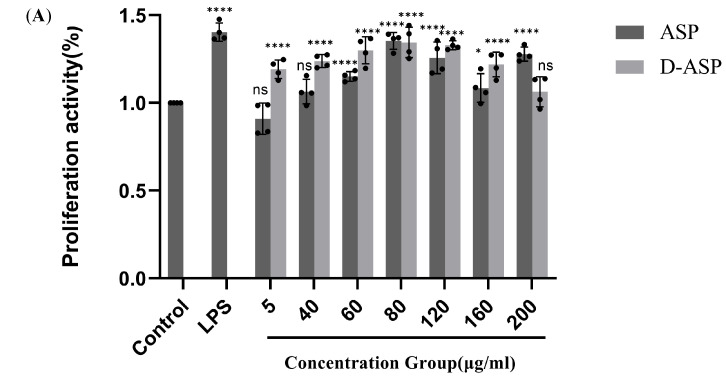

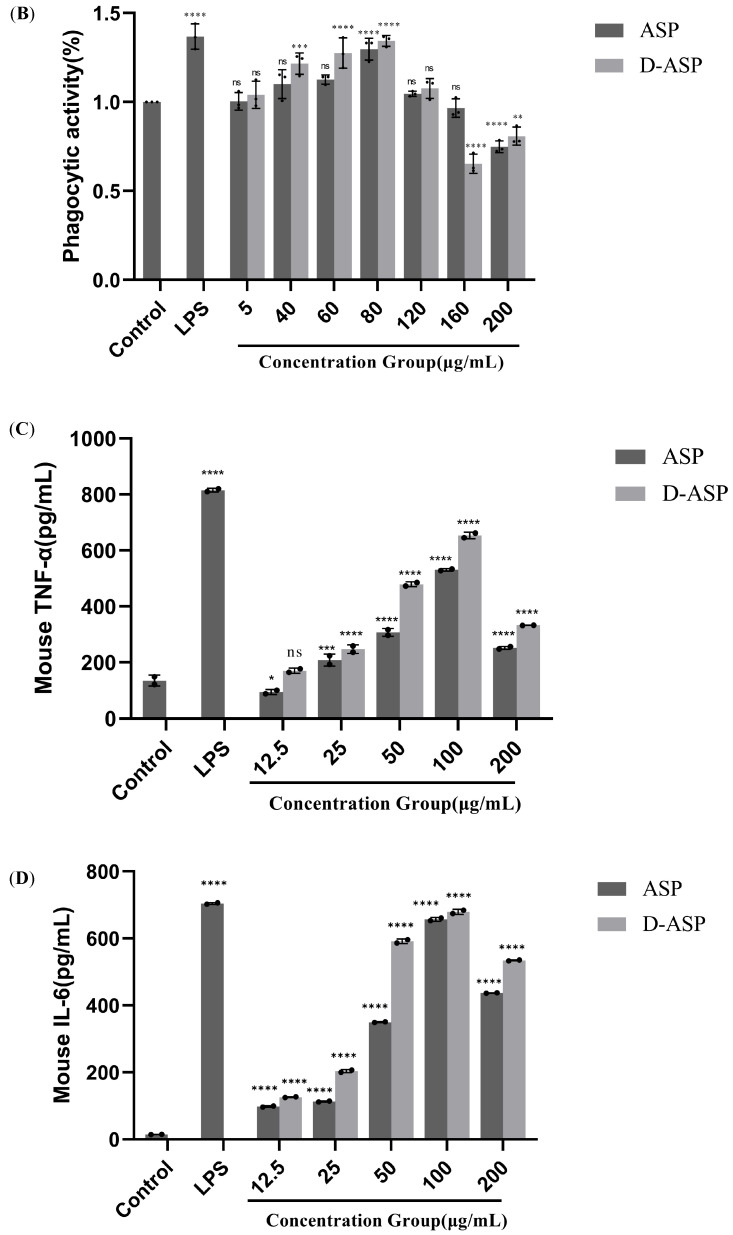

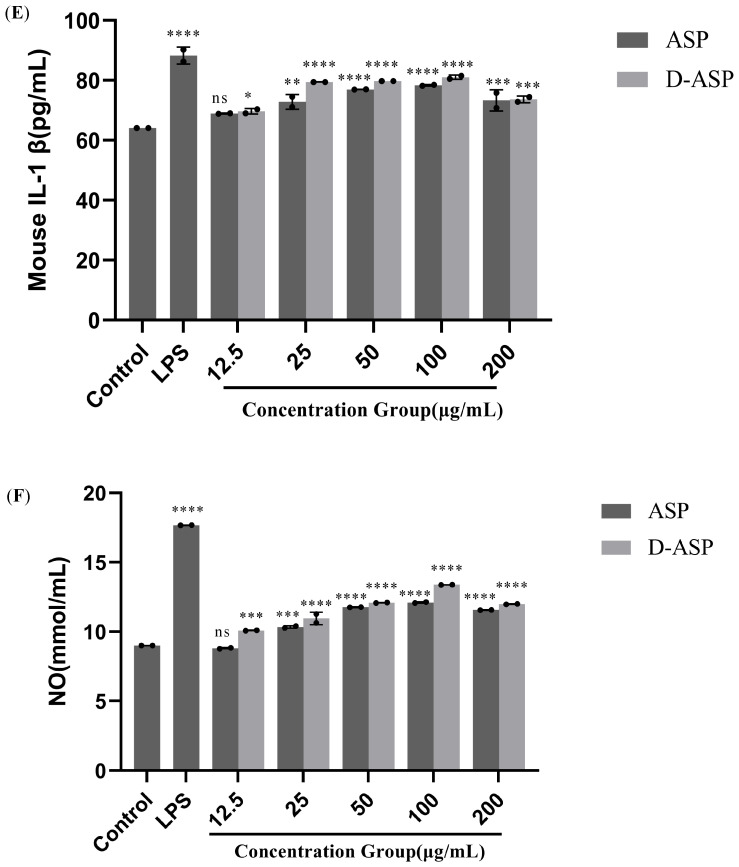
Immunomodulatory activity of ASP and D-ASP polysaccharides on RAW 264.7 cells. RAW 264.7 cells were cultured in the presence of sample polysaccharides at different concentrations for 24 h. Macrophage proliferation activity (**A**) and phagocytic activity (**B**). The culture supernatant collected was assayed for the levels of TNF-α (**C**), IL-6 (**D**), IL-1β (**E**), and NO secretion (**F**). LPS (2.5 µg/mL) was used as a positive control, and all experimental group were compared with black control. * *p* < 0.05, ** *p* < 0.01, *** *p* < 0.001, and **** *p* < 0.0001 versus cell control. ns: non-significant with respect to control.

Extensive research was conducted to assess the immunostimulatory potential of ASP and D-ASP on RAW264.7 cells. As illustrated in Figure 9A, neither ASP nor D-ASP exhibited cytotoxic effects on macrophages at concentrations ranging from 5 to 200 µg/mL. Both compounds demonstrated comparable effectiveness in promoting macrophage proliferation at lower doses, particularly at 80 µg/mL, relative to the positive control, LPS. Notably, after digestion, D-ASP continued to display a robust capacity to promote macrophage growth. At 80 µg/mL, D-ASP achieved the highest cell proliferation index, reaching 135.02% ± 8.39%, which was significantly higher than that of the control group (*p* < 0.001).

Macrophages’ phagocytosis, which maintains homeostasis, is crucial for generic immunity and specialized immunological responses [28]. Macrophages play a critical role in pathogen clearance, tissue healing, and tumor detection [29]. As illustrated in Figure 9B, ASP and D-ASP significantly enhanced macrophage phagocytic capability in a dose-dependent manner between 5 and 80 µg/mL compared with the control group. ASP and D-ASP illustrated the highest macrophage phagocytic efficiency at 80 µg/mL, up to 129.6% and 134.4%, respectively, but a little lower than the positive control LPS 136.8% (*p* < 0.0001). At 120 µg/mL, neither polysaccharide showed a significant boost in phagocytic activity (*p* > 0.05). However, at concentrations of 160–200 µg/mL, both ASP and D-ASP tended to inhibit cellular phagocytosis.

Activated macrophages release various cytokines, including TNF-α, IL-1β, and IL-6, which regulate cell differentiation, proliferation, apoptosis and death, thereby maintaining physiological homeostasis. IL-1β and TNF-a can stimulate the proliferation and differentiation of T and B cells [30], while IL-6 is important for B cell differentiation and maturation into antibody-producing cells [31]. Figure 9C–E depict the impact of ASP and D-ASP on the release of the critical immune factors involving TNF-α, IL-6, and IL-1β secretion on RAW 264.7 cells. As shown in Figure 9C–E, treatment with ASP and D-ASP promoted the production of IL-1β, IL-6, and TNF-a in RAW264.7 cells in a dose-dependent manner (12.5–100 µg/mL) compared with the control. The highest secretion levels of these cytokines were observed at 100 μg/mL. Compared with the control, the secretion levels of the digestion polysaccharide D-ASP were slightly higher than the natural ASP.

NO plays a pivotal function in the regulation of inflammation and immunity by effectively suppressing the proliferation of tumor cells and harmful bacteria. The amount of NO secreted by macrophages serves as an indirect indicator of cellular activation [32]. Proper NO generation promotes the immune response against pathogens and foreign substances. As shown in Figure 9F, both polysaccharide samples significantly promoted NO release compared with controls at 12.5–200 µg/mL (*p* < 0.0001), which was expectedly the lowest dose of ASP. The release quantity first increased in a dose-dependent manner at the range of 12.5–100 µg/mL and subsequently dropped at the highest dose. The promotion effect was the most potent at 100 µg/mL, at which concentration the NO content reached 12.1 and 13.4 µg/mL, respectively, and was lower than the positive control group at 17.7 µg/mL. These results demonstrate that both ASP and its digestion product D-ASP can activate RAW264.7 cells and induce the release of NO, TNF-α, IL-1β, and IL-6, indicating potential immunomodulatory potential.

## 3. Discussion

Polysaccharides are among the most abundant biological resources on the planet, making them essential contributors to the field of biodiversity. Zicao species, widely utilized in Chinese herbal medicine, are perennial plants known for their significant medicinal properties. In this research paper, the pectic polysaccharide ASP was extracted and purified from the root of *Arnebia szechenyi* Kanitz (Mongolia Zicao), and its structural characteristics and biological activities were subsequently evaluated. The molecular weight of ASP (18.4 kDa) was higher than that of the ARP polysaccharide (12.3 kDa) from *Arnebia euchroma* (Royle) Johnst (Xinjiang Zicao), and the sugar composition of the two polysaccharides mainly comprised Gal, Ara, Glu, and GalA. However, ASP had a higher proportion of GalA. ARP was likely an arabinogalactan (AG), which was primarily characterized by a backbone composed of Galp residues linked by 1,6 connections and Ara residues linked by either 1,5 or 1,3 connections [6]. ASP comprised the HG domain with the main backbone of 4)-GalAp-(1→, →4)-Galp-(1→, and →5)-Araf-(1→ residues linkages. The structural results showed that while the monosaccharide of two polysaccharides extracted from different Zicao species was identical, the linkages of sugar residues differed significantly. Both ASP and ARP polysaccharides strongly stimulated immunomodulatory activities, as evidenced by the increased secretion of cytokines, such as TNF-α, IL-6, IL-1β, and NO in vitro.

Pectin, a class of complex acid heteropolysaccharides commonly found in traditional Chinese medicine, can be categorized into four types, including homogalacturonan (HG), two types of rhamnogalacturonan (RG-I and RG-II), and xylogalacturonan, based on differences in sugar composition and glycosidic linkages [33,34]. RG-I can be distinguished from other domains by the ratio of Rha/GalA, which ranges from 0.05 to 1. HG is a linear chain of 1,4-linked a-d-galactopyranosyluronic acid (GalpA) residues, in which some carboxyl groups are methylated. These findings highlight that the ASP is a typical pectin-type polysaccharide rich in HG domains. An in vitro study on the effect of the HG domain on macrophage immune regulatory activity focused on inflammatory factors such as NO, TNF-α, IL-1β, and IL-10 [35]. For instance, Wang et al. isolated eight fractions from *M. alba* L fruit and one acidic fraction FPA-5, with a high GalA content (64.80%), was identified as possessing an HG domain. FPA-5 markedly induced the activation of macrophages in vitro and is considered as a potent immunostimulator [36].

Enzymatic hydrolysis is regarded as an excellent approach for investigating the structure of polysaccharides. In comparison to typical acid hydrolysis, which is non-selective, it may result in an inadequate or excessive destruction of particular glycosidic linkages, preventing the full resolution of complex structures. Enzymatic hydrolysis may selectively break designated glycosidic linkages, yielding more precise fragments and minimizing the drawbacks associated with acid hydrolysis. This technique offers high specificity and represents an innovative strategy for elucidating polysaccharide structures. We further investigated the relationship of the structure, character, and biological activity of ASP by applying enzyme digestion. We found that enzyme treatment cleaved the surface branches (Ara and Glu), which let to enhanced immune and antioxidant activities. The optimization of those activities is speculated to be possibly related to slight reductions in molecular weight, changes in the forms and ratios of monosaccharides, or subtle alterations in the spatial structure induced by enzymatic hydrolysis. For example, Kim et al. isolated a crude polysaccharide fraction (HCP) from HCE with a molecular weight of 86.6 kDa, which was primarily composed of GalA and identified as a typical HG-type pectin polysaccharide. Using endo-polygalacturonase to degrade the HG region, it produced an enzymatic hydrolysate (HCPE) with average molecular weights of 11.1 and 83.6 kDa. In vitro, HCPE strongly induced the secretion of immunostimulatory factors (including IL-6, GM-CSF, and IL-10), as well as PP-mediated BMC proliferation, compared with HCP [37]. Obtaining bioactive polysaccharide or oligosaccharide fragments via targeted enzymolysis and other advanced techniques remains challenging but is crucial for an in-depth understanding of structure–activity relationships.

## 4. Materials and Methods

### 4.1. Materials and Reagents

*Arnebia szechenyi* Kanitz (Mongolia Zicao) was collected from Wuhai City, Inner Mongolia province, China, in the summer of 2020 by our laboratory (Supplementary S1). The botanical specimens of *Arnebia szechenyi* Kanitz were processed and identified by Professor Qu from the College of Pharmacy at Inner Mongolia Medical University (Supplementary S2).

Roots were air dried, crushed, and sifted through a 600 μm sieve. Dextran standards (MW size 11,600, 23,800, 48,500, 80,900, 148,000, 409,800, and 667,800 Da), standard monosaccharides, DEAE-52 cellulose, Sephadex G-100, and all chemical reagents were purchased from Sigma-Aldrich Chemical Co., Ltd. (St. Louis, MO, USA). Dialysis membranes (MWCO size 36, 8–14 kDa) were obtained from Wako (Tokyo, Japan). The glucose standard was provided by the National Institutes for Food and Drug Control (GZDD-0114). Cell-counting kit-8 and all ELISA kits were purchased from Dakewei Bio-engineering Co., Ltd. (Beijing, China). Enzyme arabinosidase was purchased from Sigma-Aldrich Chemical Co., Ltd. (St. Louis, MO, USA). The murine monocytic macrophage cell line RAW264.7 (Cat No. SCSP-5036) was obtained from the Cell Bank of the Chinese Academy of Sciences (Shanghai, China).

### 4.2. Extraction and Purification of Polysaccharides

The crude ASP polysaccharide was extracted following the method outlined by our previous report [6] with slight adjustments. Specifically, 50 g of finely ground herb was accurately measured, thoroughly mashed, and placed in a 1000 mL round-bottom flask. Water was added to the herb in a 1:10 ratio (herb:water = 1:10), and the mixture was stirred evenly using magnetic force. The extraction was carried out at 100 °C for 1 h, which was followed by filtration. This extraction process was repeated three times with the resulting filtrates combined and concentrated to 200 mL. Ethanol was added to the concentrated filtrates to achieve a final concentration of 80%, and the mixture was left to stand at room temperature overnight to induce precipitation. The resulting precipitate was collected by centrifugation at 6000 rpm for 15 min at 4 °C, which was followed by washing three times with anhydrous ethanol and acetone ether. The precipitate was then freeze-dried to obtain the water-soluble crude polysaccharides. Deproteinization was carried out using the Sevag method. The crude polysaccharide was separated and purified by DEAE-52 cellulose column chromatography (60 cm × 4.0 cm) eluted with deionized water and graded concentrations of NaCl solution (0.1 M, 0.2 M and 0.3 M) at 2 mL/min, and the eluent fractions were collected for 6 mL/tube using an automatic collector and analyzed by the phenol-sulfuric acid method. Peak 2 was further applied on the Sephadex G-100 column (110 cm × 2.4 cm) eluted with distilled water at 0.4 mL/min. Polysaccharide and protein contents were assayed using the phenol–sulfuric acid method (with glucose as the standard) [38] and the BCA method (with BSA as the standard, Thermo Fisher, Waltham, MA, USA), respectively.

### 4.3. Enzymatic Digestion of ASP Polysaccharide

An enzymatic degradation of polysaccharide is conducive to improve the solubility and facilitate the study of conformational-biological activities and their relationships. The enzymatic degradation methodology was performed as follows. First, 0.1 g of ASP polysaccharide sample was added to 2 mg of arabinosidase and 100 mL of a buffer solution (optimal pH 3.6–4.0), and the sample was completely mixed and dissolved. The digesting reaction was conducted at 50 °C for 24 h, deactivated at 100 °C for 10 min, and centrifugated at 4500 r/min for 20 min for impurity removal. Then, 67.2 mg of the digestion sample (D-ASP) was obtained after dialyzing (Size 36, MWCO size > 8000 Da) with ultrapure water for 48 h and freeze-dried.

### 4.4. Molecular Weight Determination

The molecular weight and purity of the polysaccharides were analyzed using high-performance gel permeation chromatography [39], combined with high-performance liquid chromatography (LC-10 A, Shimadzu, Kyoto, Japan), utilizing BRT102 and 104 Gel Columns (8 mm × 300 mm) from Borui Saccharide, Biotech. Co., Ltd. (Yangzhou, China), with an exclusion range of 0.5–400 kDa. The elution process was conducted using a 0.2 M NaCl solution at a flow rate of 0.8 mL/min with detection performed at 40 °C by a refractive index detector (Shimadzu RI-10A). Both ASP and D-ASP samples were prepared at 7 mg/mL and centrifuged at 12,000 rpm for 10 min. The supernatant was filtered using a 0.45 μm microporous filter, and 20 μL of the filtrate was injected for analysis. A calibration curve was generated using a series of dextran standards with molecular weights ranging from 11,600 to 667,800 Da.

### 4.5. Monosaccharide Composition Identification

ASP and D-ASP (4 mg) were subjected to hydrolysis using 2 M trifluoroacetic acid at 120 °C for 3 h in a sealed tube. First, 200 μL of the resulting solution was collected and evaporated to dryness under nitrogen gas after hydrolysis. The dried residue was resuspended in 1 mL of water and vortexed. The supernatant was obtained by centrifugation at 12,000 rpm for 5 min and then subjected to ion chromatography analysis.

### 4.6. Ion Chromatography

Ion chromatography was conducted on an ICS system (Thermo Fisher) equipped with a Dionex Carbopac TMPA20 (3 × 150 mm) anion-exchange column and an electrochemical detector. The mobile phases were phase A, distilled water; and phase B, 250 mM NaOH:50 mM NaOH with 500 mM sodium acetate. The analysis conditions were set with a flow rate of 0.3 mL/min, an injection volume of 5 μL and a column temperature of 30 °C [40].

### 4.7. Ultraviolet–Visible (UV-Vis) Spectroscopy Analysis

First, 1 mg/mL of the ASP and D-ASP solutions in distilled water were scanned using a UV-Vis spectrophotometer (P9, MAPADA, Shanghai, China). The instrument was set to scan between 190 and 400 nm, focusing on detecting wavelengths within the range of 260 to 280 nm.

### 4.8. Infrared Spectroscopy (FT-IR) Analysis

The FT-IR spectra of ASP and D-ASP, ground with KBr in a 1:100 ratio, were obtained using an IR Affinity Fourier Transform Infrared Spectrometer (Shimadzu, Japan). The measurements were carried out via the KBr-disk method, with a resolution of 0.1 cm^−1^, over a scanning range from 4000 cm^−1^ to 400 cm^−1^.

### 4.9. Nuclear Magnetic Resonance (NMR) Spectroscopy

A solution of 30 mg of ASP was treated three times with deuterium oxide (D_2_O) and dissolved in 0.6 mL, using 3-(trimethylsilyl)-1-propanesulfonic acid sodium salt as the internal standard. Both one-dimensional and two-dimensional ^1^H NMR, ^13^C NMR, COSY, HSQC, HMBC and NOESY spectra were acquired over a two-day period at 40 °C using a Bruker Avance III 600 NMR spectrometer (Beijing, China). The data were analyzed by MestReNova software [40].

### 4.10. Methylation Analysis

Samples underwent a series of processes including methylation, hydrolysis, reduction and acetylation, which was followed by analysis via gas chromatography–mass spectrometry (GC-MS). The results were compared against standard mass spectra of glycosidic linkages. The polysaccharide sample was reduced using an automated carboxyl group reduction instrument (Yangzhou Borui Saccharide Biotech Led Co., Ltd., BR-HYY-001) (Yangzhou, China) [41].

Initially, an 80 mg sample of ASP was placed in a beaker and dissolved in distilled water, which was followed by adding the activator, N-(3-dimethylaminopropyl)-N′-ethylcarbodiimide hydrochloride. And the reaction was then carried out at pH 4.8 for 3 h, which was followed by an adjustment to pH 6.8 for another 2 h. Afterward, the sample was concentrated, dialyzed, reduced 3–5 times, and then freeze-dried. The resulting methylation product was prepared for subsequent analysis. Next, the methylated polysaccharide underwent hydrolysis with 2 M trifluoroacetic acid for 90 min, which was followed by reduction with sodium borohydride for 8 h. It was then acetylated using acetic anhydride at 100 °C for 1 h, as previously outlined [6]. Finally, the processed samples were analyzed using a GC-MS system (GCMS-QP, 2010, Shimadzu, Japan).

GC-MS conditions were as follows: RXI-5 SIL MS column (30 × 0.25 × 0.25) with a programmed temperature increase starting at 120 °C, rising at 3 °C/min up to 250 °C, and held for 5 min. The inlet and detector temperatures were set to 250 °C, and helium was used as the carrier gas with a flow rate of 1 mL/min.

### 4.11. Determination of a Triple-Helix Structure

The spatial structure of ASP and D-ASP was detected by the Congo red method [42], which detects the presence of polysaccharide triple helices by examining the red shift in the maximum absorption wavelength (λ_max_). First, 1 mg ASP and D-ASP polysaccharides were dissolved in 1 mL of deionized water. And then 2 mL of Congo red solution (80 μM) was added into the polysaccharide solution. The wavelength shift corresponding to λ_max_ of the resulting complex was measured using a UV-Vis spectrophotometer (P9, MAPADA, Shanghai, China) across different sodium hydroxide concentrations (0, 0.1, 0.2, 0.3, 0.4, and 0.5 M) within the 400–600 nm range.

### 4.12. Scanning Electron Microscopy

A thin layer of gold was applied to the polysaccharide sample using a sputter coater, and the morphology of ASP and D-ASP was examined by a Hitachi S-4800 scanning electron microscope (Tokyo, Japan), operating at 10 kV at various magnifications.

### 4.13. Antioxidant Activity of the ASP Polysaccharide and the Digestion Product D-ASP

#### 4.13.1. 2,2-Diphenyl-1-picrylhydrazyl (DPPH*) Radical Scavenging Activity Assay

The 2,2-diphenyl-1-picrylhydrazyl (DPPH•) assay is commonly employed as an initial screening method to assess antioxidant activity due to the high stability of its nitrogen [43]. In this experiment, 100 µL of ASP and D-ASP samples, and ascorbic acid (ASA) at varying concentrations (0.02–0.2 mg/mL), were mixed with 100 µL of DPPH–ethanol solution (1 × 10^−5^ g/mL) and incubated in the dark at 37 °C for 30 min. The absorbance was recorded at 517 nm. ASA was used as the positive control. The scavenging activity was determined by the following equation:

Scavenging activity (%) = 1 − (A1 − A2)/A0 × 100%(1)

where A1 is a mixture of 100 μL of the polysaccharide samples and 100 μL DPPH solution; A2 is the blank (polysaccharide samples and 50% ethanol solution), while A0 is the control (DPPH and 50% ethanol solution).

The experiment was conducted in triplicate with each sample measured in duplicate.

#### 4.13.2. Superoxide Anion Radical (O^2•−^) Scavenging Activity

The superoxide anion radical is generated through the transfer of an electron to molecular oxygen (O_2_). Although O^2•−^ is a weak oxidizing agent, it can react with H_2_O_2_ to form highly reactive hydroxyl radicals (OH•) [44]. Since superoxide anions are commonly produced during various cellular metabolic processes, the scavenging of O^2•−^ radicals is often used to evaluate the antioxidant activity of polysaccharides, helping to predict their potential in vivo effects.

In the P-TEMED system, superoxide anions are generated, which then react with hydroxylamine hydrochloride to form nitrite (NO^2−^). This nitrite subsequently reacts with p-aminobenzenesulfonic acid and α-naphthylamine to produce a red azo compound with a distinct absorption peak at 530 nm. The scavenging activity of superoxide anions was assessed using the protocol outlined in the superoxide anion scavenging kit (micro method). In brief, 10 mL of Tris-HCl (TEMED) and 40 mL of ammonium persulfate were added to both the control and sample tubes. Additionally, 25 mL of water was added to the control tube alone. The mixtures were incubated for 1 min at 25 °C. After incubation, 25 mL of a polysaccharide solution and a positive control solution (ASA, 0.02–0.5 mg/mL) were added to the sample tube. Both tubes were then supplemented with 50 mL of hydroxylamine hydrochloride solution and incubated at 37 °C for 30 min. Finally, 50 mL of p-aminobenzene sulfonic acid and α-naphthylamine in acetic acid were added, allowing color development at 37 °C for 20 min. The absorbance (A) was measured at 530 nm, and the superoxide radical scavenging activity was calculated by formula 1 (Supplementary S3).

#### 4.13.3. Hydroxyl Radical (OH•) Scavenging Ability

Hydroxyl radicals (OH•) can be produced from superoxide anion (O^2•−^) and hydrogen peroxide (H_2_O_2_). These radicals are highly reactive species, meaning any compound can display scavenging activity toward OH•, thereby acting as antioxidants [45]. This reactivity limits the extrapolation of in vitro findings using OH• to in vivo antioxidant effects. Nevertheless, since OH• forms from O^2•−^ and H_2_O_2_ through interactions with iron or copper ions, experiments involving OH• are valuable for understanding antioxidant mechanisms. Hydroxyl radicals are generated through the Fenton reaction, where H_2_O_2_ and Fe^2+^ react to form OH•, oxidizing Fe^2+^ to Fe^3+^ and reducing absorbance at 536 nm. The extent of inhibition of this absorbance decrease reflects the sample’s hydroxyl radical scavenging ability [45].

The hydroxyl radical scavenging assay was evaluated following the instructions of the hydroxyl radical scavenging kit, utilizing a manual micro-method. A stock solution was prepared using a mixture of phenanthroline–ethanol solution, Na_2_HPO_4_·12H_2_O–NaH_2_PO_4_·2H_2_O buffer, and FeSO_4_·7H_2_O. This working solution (250 μL) was added to blank, control, and sample tubes. In the blank tube, 100 μL of distilled water was added, while in the control tube, 50 μL of H_2_O_2_ and 50 μL of distilled water were used. The sample tube contained 50 μL of sample solution (ranging from 2–10 mg/mL ASP and D-ASP or positive control) and 50 μL of H_2_O_2_. After mixing, the tubes were incubated at 37 °C for 60 min, and absorbance was measured at 536 nm. The scavenging activity of the hydroxyl radicals was calculated using formula 2 (Supplementary S3).

### 4.14. Immunomodulatory Activity Assay

#### 4.14.1. RAW 264.7 Macrophage Proliferation

Cell-counting kit-8 assay was employed to assess cell proliferation [46]. Logarithmically growing cells were diluted to a concentration of 1 × 10^5^ cells/mL, and 100 μL of this suspension was added to each well of a 96-well plate. To minimize edge effects, 100 μL of phosphate-buffered saline (PBS) buffer was added to the wells along the perimeter of the plate. The plate was incubated at 37 °C in 5% CO_2_ for 24 h. For the experimental group, ASP and D-ASP solutions were added to achieve final concentrations of 5, 40, 60, 80, and 120 μg/mL. Each concentration was tested in triplicate wells, and the plate was incubated for 36 h. The control group received no drug treatment, while the positive control group was treated with lipopolysaccharide (LPS) at a final concentration of 2.5 μg/mL. After 36 h, the supernatants were removed, and 100 μL of medium containing 10% cell-counting kit-8 solution was added. The plate was incubated for one additional hour, after which the optical density at 450 nm was measured using a microplate reader. The cell proliferation rate was then calculated using the following formula 3 (Supplementary S4).

#### 4.14.2. Nitric Oxide (NO) Cytokine Production

Macrophage cell suspensions (500 μL) were seeded in a 48-well plate at a density of 1 × 10^5^ cells/mL using RPMI 1640 medium. For the positive group, 100 μL of suspension was treated with 2.5 μg/mL LPS, while the experimental group received 12.5–200 μg/mL polysaccharide. The final volume in each well was adjusted to 200 μL. Following a 48 h incubation period at 37 °C in 5% CO_2_, supernatants from each well were collected for nitric oxide (NO) measurement using the Griess assay according to the NO kit protocol. All experiments were performed in triplicate. The NO amount was calculated by the standard curve equation: y = −0.000004x^2^ +0.0095x + 0.0008R^2^ = 0.999.

#### 4.14.3. TNF-α, IL-1β, and IL-6 Cytokine Production

RAW 264.7 cells were plated into 96-well plates at a density of 1 × 10^6^ cells per well and treated with ASP and D-ASP polysaccharides at varying concentrations (12.5–200 μg/mL) for 48 h. Cells treated with medium alone served as the blank control, while cells treated with 2.5 μg/mL LPS were used as the positive. Following incubation, the culture supernatants were collected to measure the levels of interleukin-6 (IL-6), tumor necrosis factor-alpha (TNF-α), and interleukin-1 beta (IL-1β) using mouse precoated ELISA kits (Dakewei Bio-engineering Co., Ltd., Shenzhen, China) as per the manufacturer’s instructions.

#### 4.14.4. The Phagocytic Activity of RAW 264.7 Cells

The phagocytic activity of RAW 264.7 macrophages was assessed using a neutral red staining method [46]. Cells in the logarithmic growth phase were prepared into a cell suspension at a density of 4 × 10^5^ cells/mL. A volume of 100 μL of the suspension was seeded into each well of a 96-well plate with 100 μL of PBS added to the peripheral wells to prevent edge effects. The plate was then incubated at 37 °C in a 5% CO_2_ atmosphere for 24 h. For controls, no treatment was applied to the negative control group, while LPS (final concentration: 2.5 μg/mL) was added to the positive control group. The experimental groups received ASP and D-ASP solutions, which were adjusted to final concentrations of 5–200 μg/mL. Each concentration was tested in triplicate wells, and cells were incubated under the same conditions for an additional 24 h. After incubation, the supernatant was discarded, and cells were washed twice with PBS following the kit instructions. Next, 100 μL of medium containing 10% neutral red solution was added and incubated for 1 h. Afterward, the cells were washed twice with PBS again, and 100 μL of cell lysate was added, which was followed by further incubation for 2 h. The optical density (OD) was measured at 540 nm using a microplate reader, and the macrophage phagocytic index was calculated using formula 4 (Supplementary S4)

### 4.15. Statistical Analysis

The experimental data are expressed as mean ± standard deviation (SD) and were analyzed using GraphPad Prism 10 (GraphPad Software, San Diego, CA, USA) statistical software and analyzed using a one-way (ANOVA) test for significant differences between groups and blanks. * *p* < 0.05, ** *p* < 0.01, and *** *p* < 0.001 compared with the control group.

## 5. Conclusions

In traditional Chinese and Mongolian medicine, medicinal Zicao is primarily employed in heat clearing and blood cooling with pharmacological benefits including anti-inflammatory, antitumor, hepatoprotective, and immunomodulatory properties. Studies on the active components of traditional treatment and new clinical uses have primarily focused on shikonin of Zicao, more detailed studies have revealed that shikonin may also play a role in immune function, whereas another immunologically active polysaccharide has frequently been overlooked. In the previous study, we found that the compositions and contents of shikonin in Xinjiang Zicao and Mongolia Zicao were close to each other, so the two species could be mixed or replaced in clinical trials. However, this paper demonstrated that two polysaccharides both boosted immunostimulatory activities in macrophage cell in vitro, but varied significantly in monosaccharide composition and primary structure, which may result in various therapeutic benefits, and they cannot be simply equated with two medicinal Zicao. This will give some scientific support for the therapeutic use of medicinal Zicao.

## Figures and Tables

**Table 1:** GC–MS analysis of the methylated product of ASP.

RT	Methylated Sugar	Mass Fragments (*m*/*z*)	Molar Ratio	Type of Linkage
16.572	2,3,5-Me3-Araf	43, 71, 87, 101, 117, 129, 145, 161	0.108	Araf-(1→
22.009	2,3-Me2-Araf	43, 71, 87, 99, 101, 117, 129, 161, 189	0.127	→5)-Araf-(1→
26.135	2-Me1-Araf	43, 58, 85, 99, 117, 127, 159, 201	0.013	→3,5)-Araf-(1→
Arabinose			0.248	
20.45	2,4-Me2-Xylp	43, 58, 85, 99, 101, 117, 127, 159, 173	0.014	→3)-Xylp-(1→
22.341	2,3-Me2-Xylp	43, 71, 87, 99, 101, 117, 129, 161, 189	0.026	→4)-Xylp-(1→
Xylose			0.040	
24.281	2,3,4,6-Me4-Glcp	43, 71, 87, 101, 117, 129, 145, 161, 205	0.036	Glcp-(1→
31.342	2,3,4-Me3-Glcp	43, 87, 99, 101, 117, 129, 161, 189, 233	0.009	→6-Glcp-(1→
34.183	2,6-Me2-Glcp	43, 87, 97, 117, 159, 185	0.030	→3,4)-Glcp-(1→
37.16	2,3-Me2-Glcp	43, 71, 85, 87, 99, 101, 117, 127, 159, 161, 201	0.042	→4,6)-Glcp-(1→
Glucose			0.117	
25.392	2,3,4,6-Me4-Galp	43, 71, 87, 101, 117, 129, 145, 161, 205	0.013	Galp-(1→
29.645	2,3,6-Me3-Galp	43, 87, 99, 101, 113, 117, 129, 131, 161, 173, 233	0.229	→4)-GalAp-(1→
30.114	2,3,6-Me3-Galp	43, 87, 99, 101, 113, 117, 129, 131, 161, 173, 233	0.128	→4)-Galp-(1→
37.659	2,3-Me2-Galp	43, 71, 85, 87, 99, 101, 117, 127, 159, 161, 201, 261	0.009	→4,6)-Galp-(1→
39.474	2,4-Me2-Galp	43, 87, 117, 129, 159, 189, 233	0.018	→3)-Galp-(1→
41.723	3-Me1-Galp	43, 87, 99, 127, 129, 189, 201, 261	0.008	→2,4,6)-Galp-(1→
Galactose			0.405	

**Table 2:** Chemical shifts of resonance in ^13^C and ^1^H NMR spectra of ASP.

No.	Type of Linkage	Proton or Carbon (^1^H/^13^C, ppm)							
		H1/C1	H2/C2	H3/C3	H4/C4	H5/C5	H6/C6		Ref.
A	Araf-(1→	5.14/107.7	4.13/76.8	3.96/81.5	4.23/82.4	3.80/61.34	---		[22]
B	→5)-Araf-(1→	5.17/107.3	4.13/82.41	4.09/76.8	4.22/84.1	3.87/68.3	---		[18]
C	→3,5)-Araf-(1→	5.24/107.1	4.30/82.5	4.09/84.3	4.13/84.3	3.90/66.5	----		[23]
D	→4)-Glcp-(1→	5.12/100.2	3.71/71.9	4.00/73.5	--	3.67/63.02	3.88/61.5		[24]
E	1→4)-GalpA-(6-OMe)-	5.03/100.5	3.62/71.53	3.82/68.2	4.29/79.0	4.53/78.6	n.d./171.0	3.88/53.03(-OMe)	[25]
F	→3or 4)-Galp-(1→	4.60/103.1	3.80/72.9	3.73/74.8	3.83/80.5	4.03/73.53	3.87/61.34		[26]
G	→4)-Xylp-(1→	4.58/103.2	3.39/72.9	4.48/73.1	3.58/75.7	3.91/63.2			[18]

## Data Availability

The original contributions presented in this study are included in the article/Supplementary Material. Further inquiries can be directed to the corresponding author(s).

## Supplementary Materials

The following supporting information can be downloaded at https://www.mdpi.com/article/10.3390/molecules30193852/s1, Supplementary S1: Photos of *Arnebia szechenyi* Kanitz (eco logical environment, flowering plant); Supplementary S2: Specimen and specimen number of *Arnebia szechenyi* Kanitz; Supplementary S3: Formulas 1 and 2; Supplementary S4: Formulas 3 and 4.

## Abbreviation

The following abbreviations are used in this manuscript:

TNF-α	Tumor necrosis factor-alpha
IL-6	Interleukin-6
IL-1β	Interleukin-1β
DEAE-52 cellulose	Diethylaminoethyl-cellulose
MWCO	Molecular weight cut-off
BSA	Bovine serum albumin
DPPH	2,2-diphenyl-1-picrylhydrazyl radical
Mw	Weight-average molecular weight
O^2•−^	Superoxide anion radical
(OH•)	Hydroxyl radical
HPGPC	High-performance gel permeation chromatography
IC	Ion chromatography
SEM	Scanning electron microscopy
IR	Infrared spectroscopy
NMR	nuclear magnetic resonance spectroscopy
GC-MS	Gas chromatography–mass spectrometry
COSY	1H–1H homonuclear correlation spectroscopy
HSQC	1H–13C heteronuclear single quantum coherence
NOESY	Nuclear Overhauser effect spectroscopy
HMBC	The long-range heteronuclear multiple bond correlation
RG-I	Rhamnogalacturonan-I
HG	Homogalacturonan
PBS	Phosphate-buffered saline
GalA	Galacturonic acid
LPS	Lipopolysaccharide
