# Supplementary material for: A Pectin Polysaccharide from Arnebia szechenyi Kanitz and Its Digestion Product: Physicochemical Properties and Immunostimulatory and Antioxidant Activities
Source: Molecules. 2025 Sep 23;30(19):3852. doi: 10.3390/molecules30193852 (PMC12525583; doi:10.3390/molecules30193852)
Supplement: Supplementary file 1 [file molecules-30-03852-s001.zip › molecules-3856517-supplementary.pdf]

Supplementary Materials

Supplementary S1.

Photos of *Arnebia szechenyi Kanitz* (ecological environment, flowering plant)

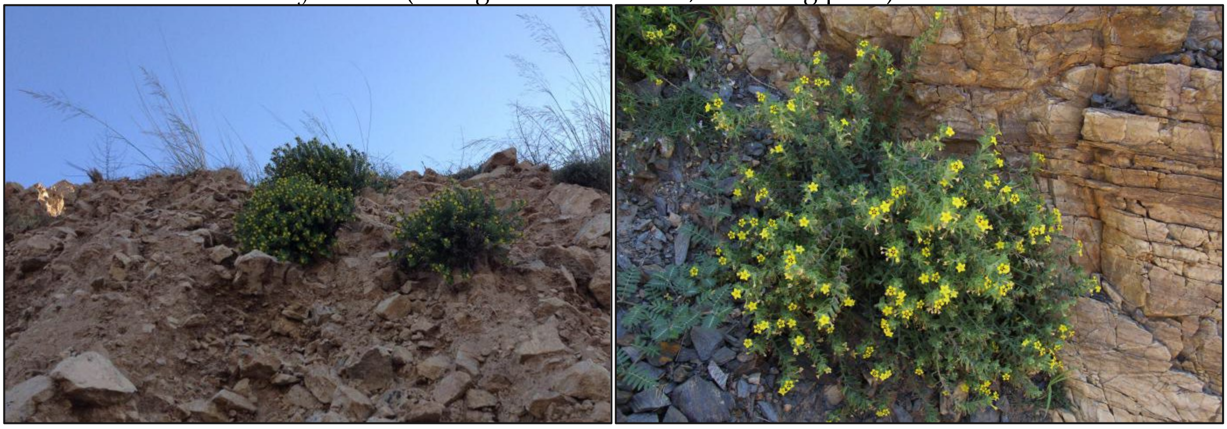

Supplementary S2.

Specimen and specimen number of *Arnebia szechenyi* Kanitz

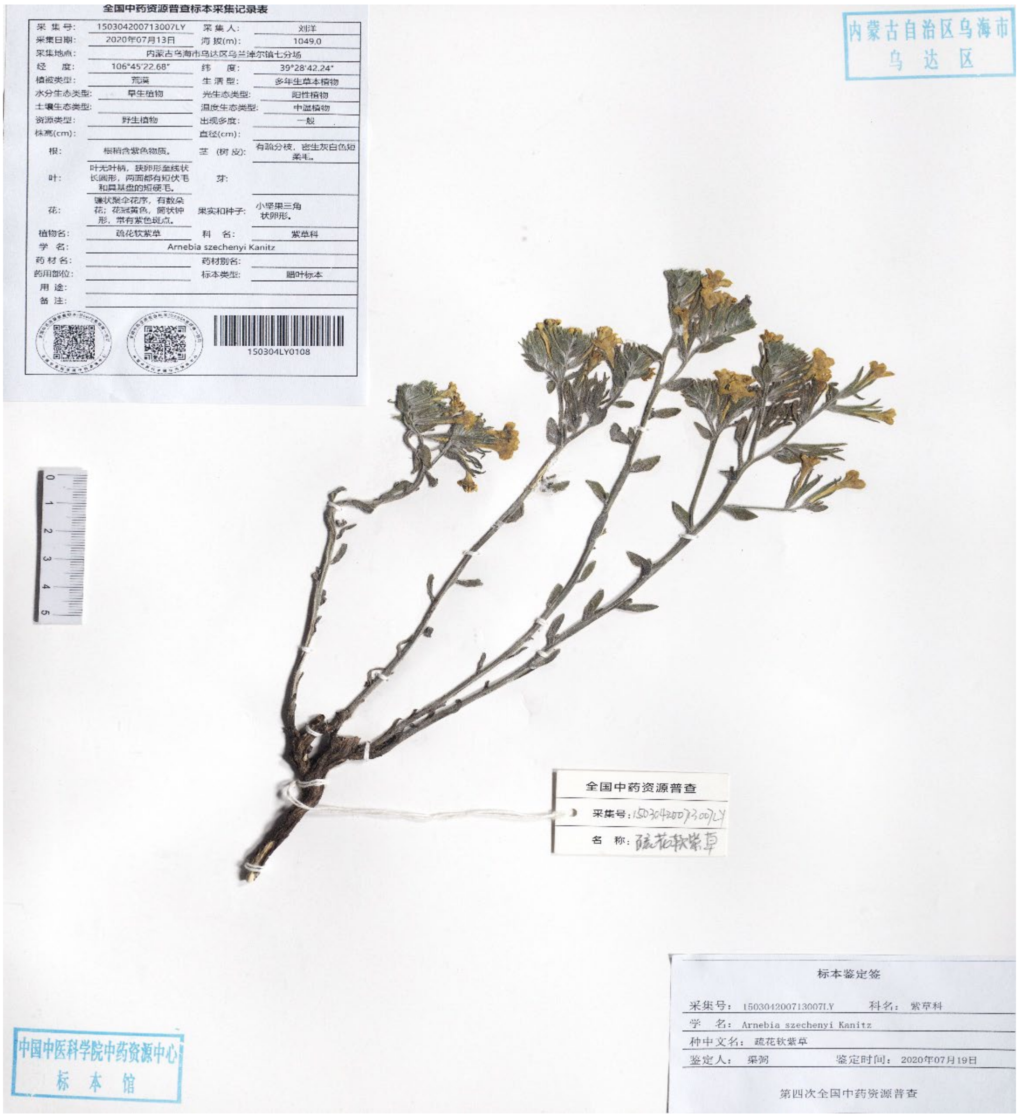

Supplementary S3.

Formula (1)

Scavenging activity (%) = $(1 - A_{sample}/A_{control}) \times 100\%$.

Where A sample is the absorbance of the sample, and $A_{control}$ is the absorbance of the control (distilled water replacing H_2O_2). The experiment was conducted three times, with each run containing duplicate samples.

Formula (2)

Scavenging activity (%) = $(A_{sample} - A_{control}) / (A_{blank} - A_{control}) \times 100\%$

Where A_{blank} is the absorbance of the blank (distilled water instead of the sample), A sample is the absorbance of the sample, and $A_{control}$ is the absorbance of the control (distilled water replacing H_2O_2). The experiment was conducted three times, with each run containing duplicate samples.

Supplementary S4.

Formula (3)

$$Cell\ viability(\%) = \frac{A1 - A0}{A2 - A0} \times 100\%$$

Formula (4)

$$\mathrm{PI}(\%) = \frac{A1}{A2} \times 100\%$$
